# Supplementary material for: miR‐31 mutants reveal continuous glial homeostasis in the adult Drosophila brain
Source: EMBO J. 2017 Mar 20;36(9):1215–26. doi: 10.15252/embj.201695861 (PMC5412881; doi:10.15252/embj.201695861)
Supplement: Supplementary file 1 — Appendix [file EMBJ-36-1215-s001.pdf]

Appendix Table of Contents

Title	Page(s)
1. Appendix figure legends	2-4
2. Appendix Figure S1	5
3. Appendix Figure S2	6
4. Appendix Figure S3	7
5. Appendix Figure S4	8
6. Appendix Figure S5	9
7. Appendix Figure S6	10
8. Appendix Figure S7	11
9. Appendix Figure S8	12
10. Appendix Figure S9	13
11. Appendix Figure S10	14

1. Appendix Figure Legends

Appendix Figure S1

(**A**) Raw graph depicting the number of anti-Repo-expressing cells in 2d, 7d and 21d post-eclosion Canton S (Ctrl) and *mir-31a* mutant (KO) brains.
(**B**) Representative confocal stack images of Figure 1B and Appendix Figure S1A. Yellow dashed lines demarcate the central brain where the cells were counted.

Appendix Figure S2

(**A**) Raw graph depicting the number of *Alrm-Gal4*-expressing, *NP577*-expressing and *NP6520*-expressing cells in 7d old post-eclosion adult brains.
(**B**) Raw graph depicting the number of *Alrm-Gal4*-expressing cells in 7d and 21d old post-eclosion adult brains.
(**C**) Representative confocal stack images of Figure 1C and Appendix Figure S2A. Yellow dashed lines demarcate the central brain where the cells were counted.

Appendix Figure S3

(**A-D**) Raw graphs depicting the number of anti-Repo-expressing glia at 7 days of age. Data was analysed using one-way Anova with post-hoc Tukey analysis (**A-D, G-H**). Error bars represent SEM. For each Gal4 driver tested, Gal4/+ was compared with the *UAS-mir-31a sponge* transgene/+ and with Gal4 directing expression of the sponge (Gal4>31a sponge). (**A**) *Repo-Gal4* (**B**) *Syb-Gal4*. (**C**) *Elav-Gal4* (**D**) *Insc-Gal4, Df(3L)H99* indicates flies carrying one copy of this deletion, to limit apoptosis in the presence of the *mir-31a* sponge or *UAS-GFP*.

(**E, F**) Raw number of anti-Repo-expressing glia following adult-specific depletion of *mir-31a.* Flies carrying *Gal80^{ts}* with *Insc-Gal4* (**E**) or *mir-31a-Gal4* (**F**) and the *UAS-mir-31a* sponge or *UAS-GFP* were raised at 18°C until adults had eclosed, and were then shifted to 29°C to allow Gal4 activity. *UAS-GFP* was used as a control. Flies were examined 7 days after Gal4 activation. Data were analysed using an unpaired two-tailed Student's t-test.

(**G, H**) Raw number of anti-Repo-expressing glia in brains at 7 days. *UAS-GFP* was used as a control for expression of *UAS-mir-31a* transgene. Ctrl: Canton S control. *31aKO/KO* indicates the homozygous mutant. Data were analysed using one-way Anova with post-hoc Tukey analysis. (**G**) *Insc-Gal4* was used to direct transgene expression. (**H**) The *mir-31-a-Gal4* allele was used to direct transgene expression. Gal4/+ indicates *mir-31-a-Gal4* allele in trans to wild-type. mir-31-a-Gal4/KO indicates the Gal4 allele in trans to the deletion allele.

Appendix Figure S4 and 5

Representative confocal stacks of the genotypes from Figure 2 and Appendix Figure S3. Yellow dashed lines demarcate the central brain where the cells were counted.

Appendix Figure S6

(**A**) Representative confocal stacks of the genotypes from Figure 2 and Appendix Figure S3. Yellow dashed lines demarcate the central brain where the cells were counted.

(**B**) Confocal stack images of brains from 1d old adult *Alrm-Gal4>UAS-Histone-RFP (*red)*, mir-31a* sensor (green). White arrowheads point to cells that are *Alrm-Gal4>UAS-Histone-RFP*$^{+}$ and *mir-31a*$^{+}$ (GFP negative).

(**C**) Higher magnification, single optical sections of (**B**). Image is of the antennal lobe. White arrowheads point to cells that are *Alrm-Gal4>UAS-Histone-RFP*$^{+}$and *mir-31a*$^{+}$ (GFP negative).

(**D**) Number of anti-Repo-expressing glia at 7 days of age post-eclosion. Glia counts are represented as a percentage of the average number of glia in central brains of the Gal4/+ controls for each panel. Data was analysed using one-way Anova with post-hoc Tukey analysis. Error bars represent SEM. Worniu-Gal4 (WorGal4) was used and WorGal4/+ was compared with the *UAS-mir-31a sponge* transgene/+ (31a sponge/+) and with Worniu-Gal4 directing expression of the sponge (WorGal4>31a sponge).

(**E**) Raw numbers of each genotype for (**D**).

Appendix Figure S7

(**A-H**) Raw number of anti-Repo-expressing glia in brains at 7 days. Data were analysed using one-way Anova with post-hoc Tukey analysis (**B, D, E G**) and unpaired student's t-test for (**A, C, F, H)**. Error bars represent SEM.

(**A**) Raw number of anti-Repo-expressing cells in *mir-31a* mutants (KO) and in mutants carrying the *Df(3L)H99* deficiency.

(**B**) The *UAS-CG16947* RNAi transgene without a Gal4 driver was used as a control. mir-31a KO/KO indicates the homozygous deletion mutant. A *UAS-GFP* transgene was used as a control for expression of the RNAi transgene with *Insc-Gal4* in the mutant background. ns: not significant. See Table EV1.

(**C**) Expression of *UAS-GFP* or *UAS-CG16947* RNAi using *Insc-Gal4* cells in an otherwise normal background.

(**D**) The *UAS-CG16947* transgene without a Gal4 driver was used as a control. *UAS-GFP* was used as a control for expression of the *UAS-CG16947* transgene with *Insc-Gal4* in an otherwise normal background.

(**E**) All samples carried one copy of the *mir-31a*-Gal4 allele. KO; GFP indicates the deletion allele and a *UAS-GFP* transgene. KO, CG16947 RNAi indicates the deletion allele and the UAS-RNAi transgene to deplete CG16947 mRNA.

(**F**) All flies carried the *mir-31a-Gal4* allele. KO indicates the deletion allele.

(**G**) The *UAS-CG16947* transgene without a Gal4 driver was used as a control. *UAS-GFP* in the mutant background was used for comparison to expression of *UAS-CG16947* transgene with *mir-31a-Gal4* in an otherwise normal background.

(**H**) Number of anti-Repo-expressing glia in 7 day post-eclosion brains from controls expressing *UAS-GFP* or *UAS-CG16947* in glia under *Repo-Gal4* control.

Appendix Figure S8

Representative confocal stacks of the genotypes from Figure 3 and Appendix Figure S7. Yellow dashed lines demarcate the central brain where the cells were counted.

Appendix Figure S9

Representative confocal stacks of the genotypes from Figure 3 and Appendix Figure S6D,E and S7. Yellow dashed lines demarcate the central brain where the cells were counted.

Appendix Figure S10

(**A**) Representative confocal stacks of the genotypes from Figure 5J, K. Yellow dashed lines demarcate the central brain where the cells were counted.

(**B**) Raw numbers of Figure 5K. Flies carrying *Repo-Gal4* and $Gal80^{ts}$ were reared at 18°C until 14d whereupon they were moved to 29°C for 2d to induce the expression of *UAS-Hid* or *UAS-GFP* as a control. Left panels: flies were examined immediately after 2 days of transgene expression. Right panels: flies were allowed to recover for 14 days at 18°C before processing. Data were analysed using an unpaired t-test (two tailed). ns: not significant. Error bars represent SEM.

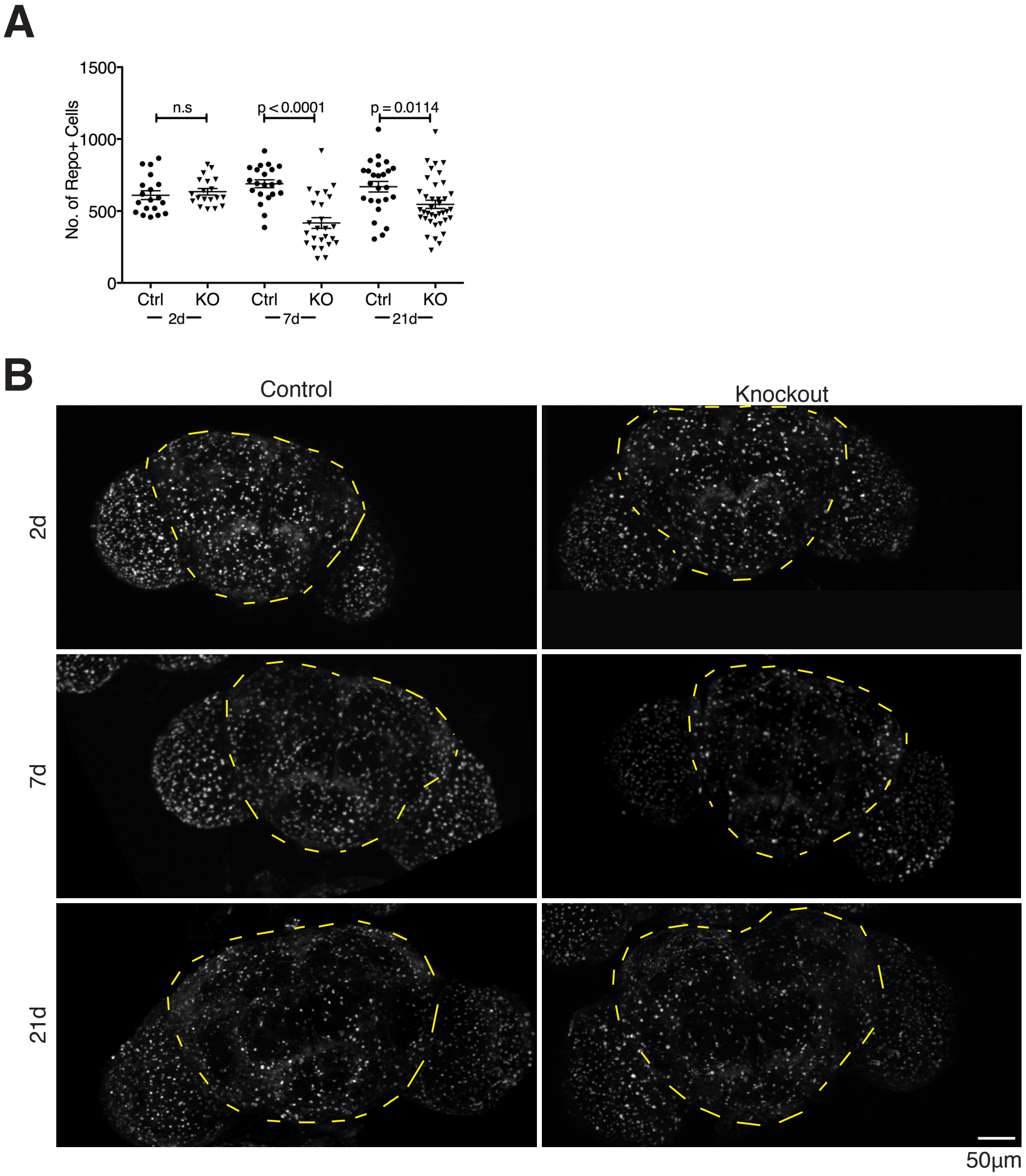

Appendix Figure S1

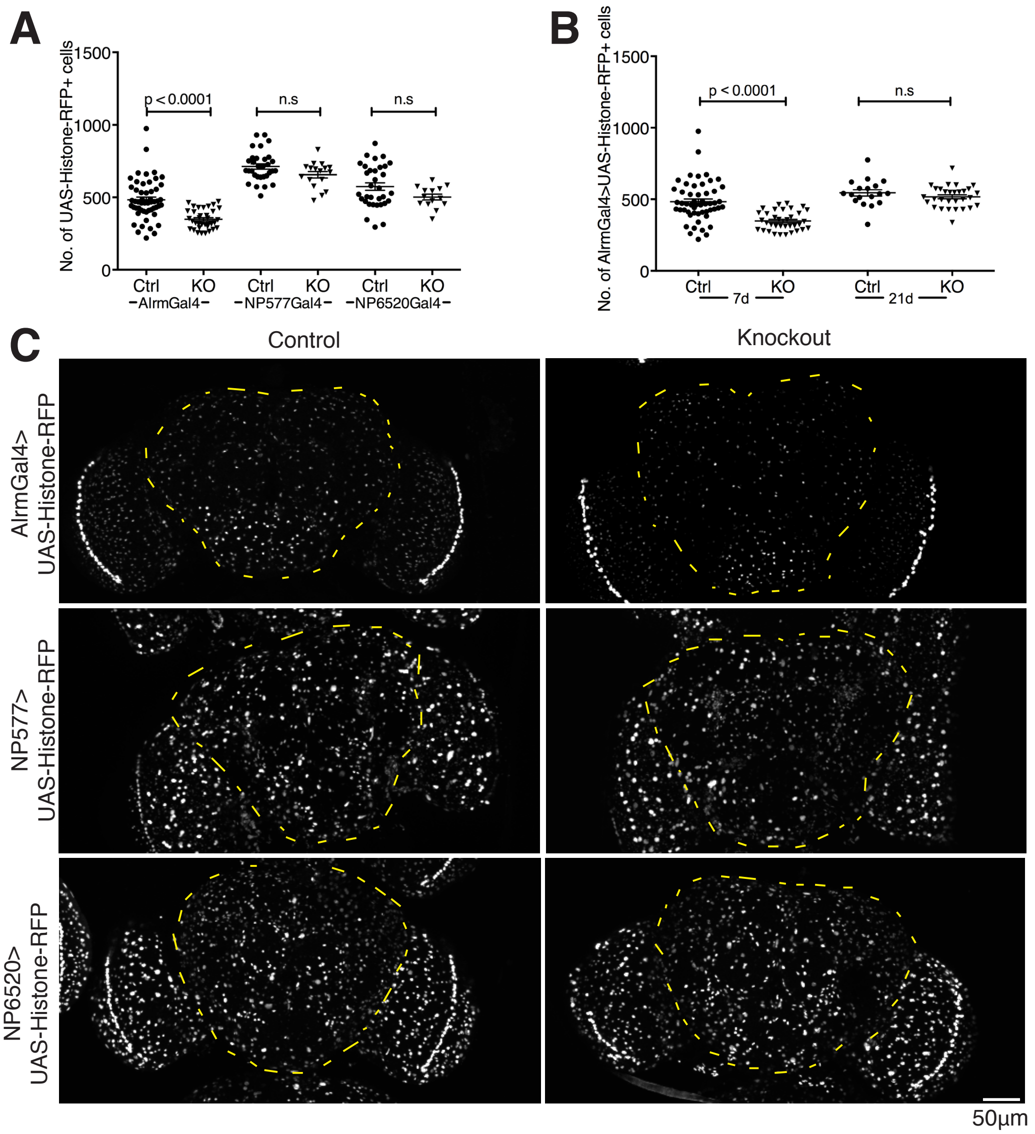

Appendix Figure S2

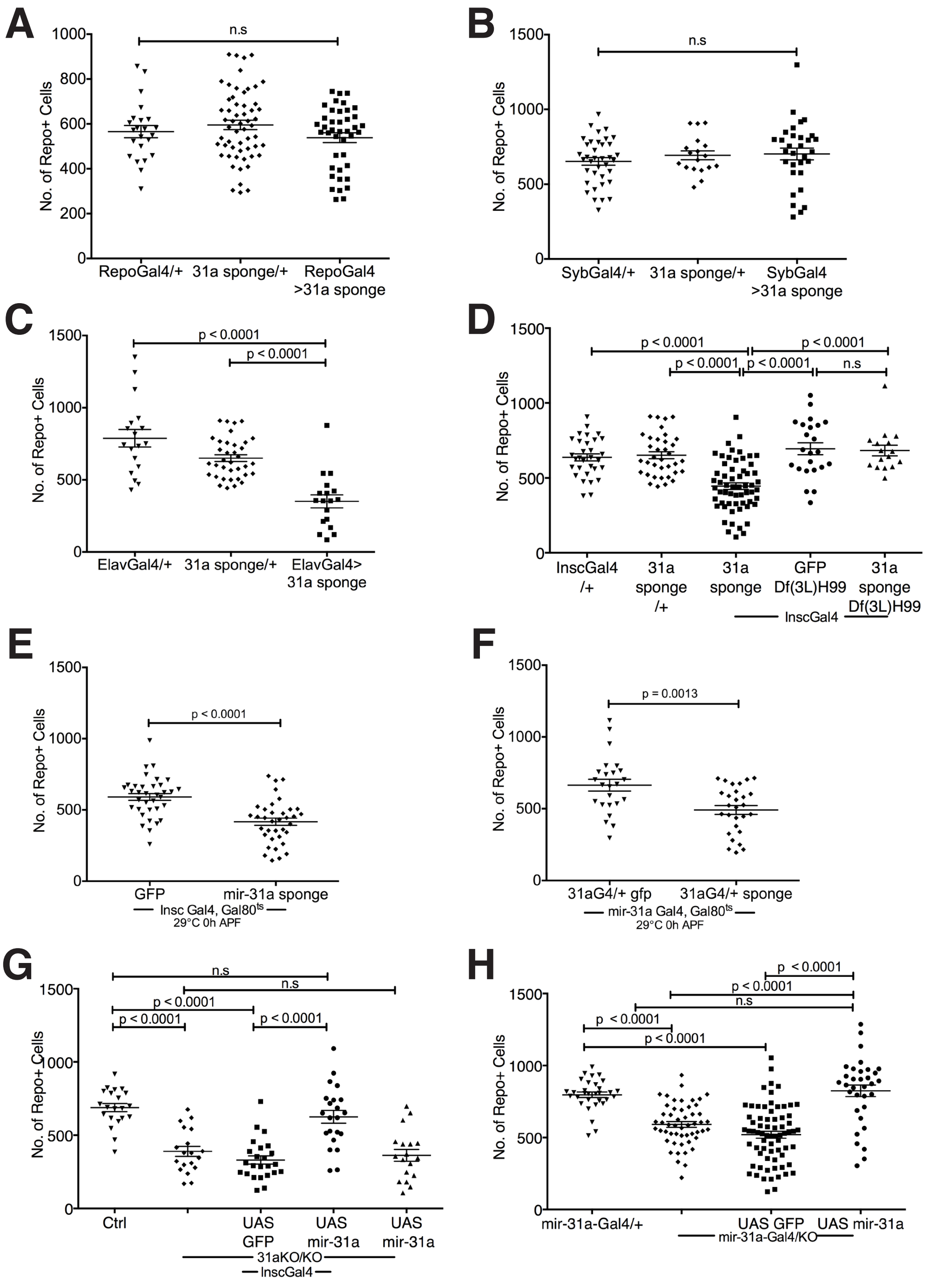

Appendix Figure S3

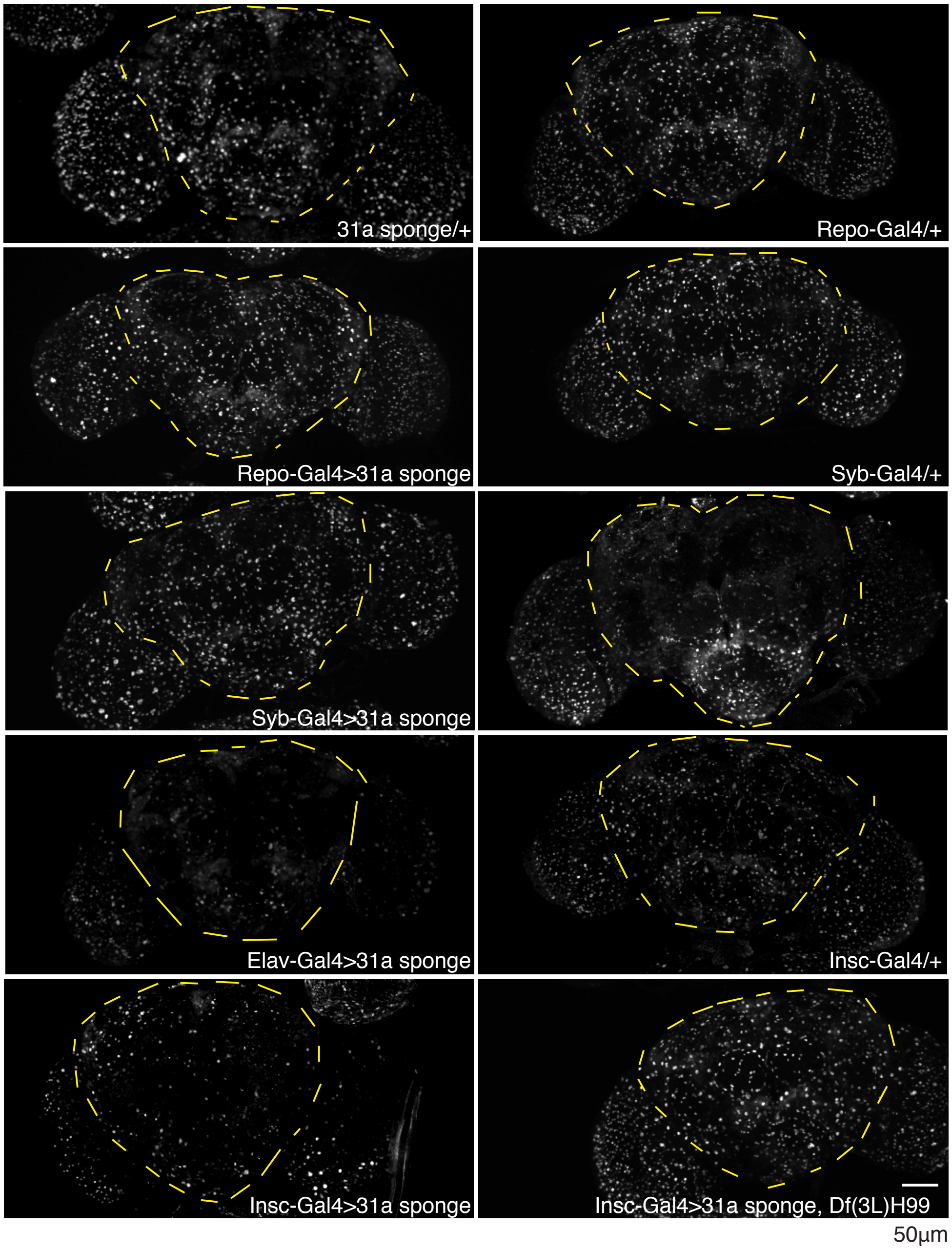

Appendix Figure S4

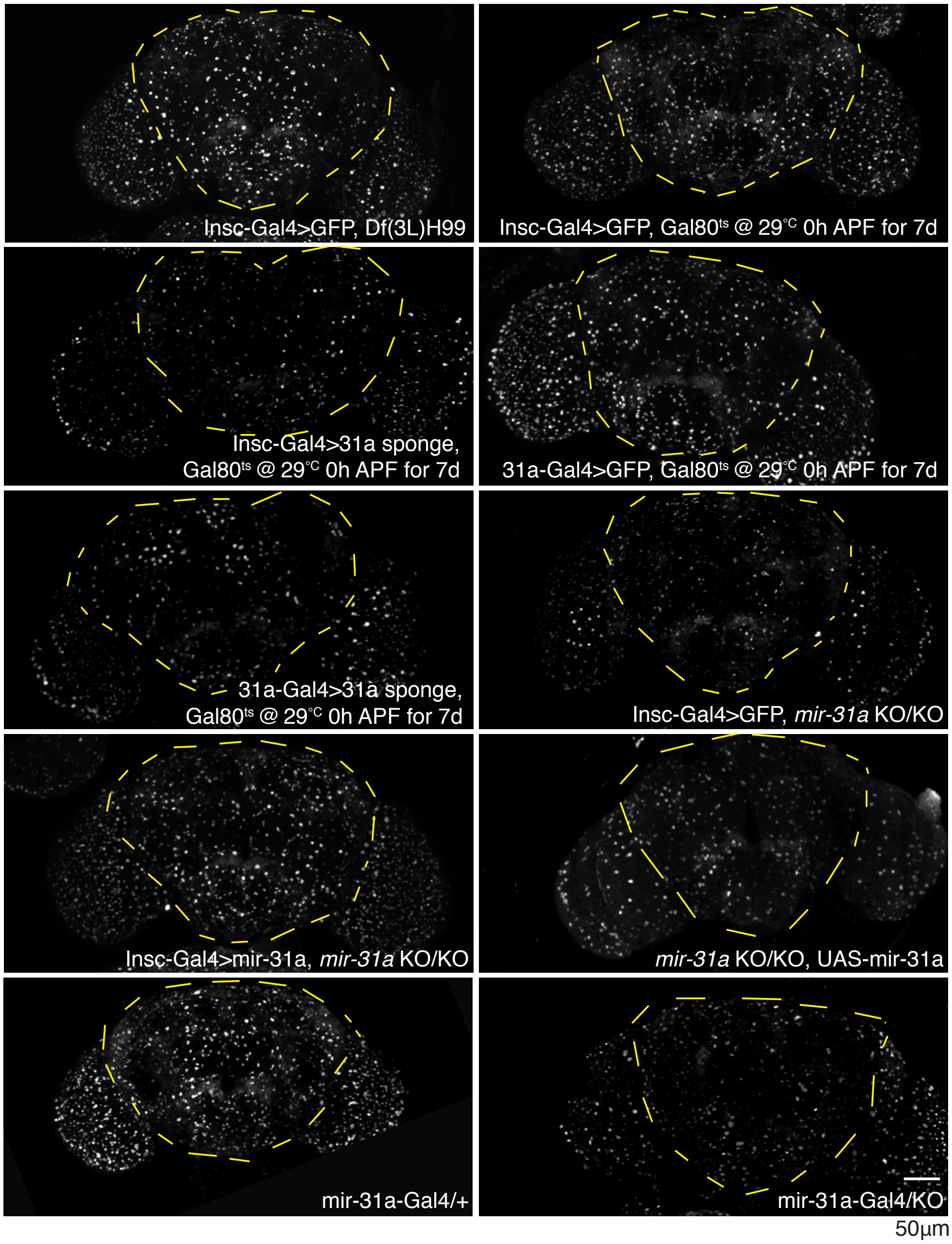

Appendix Figure S5

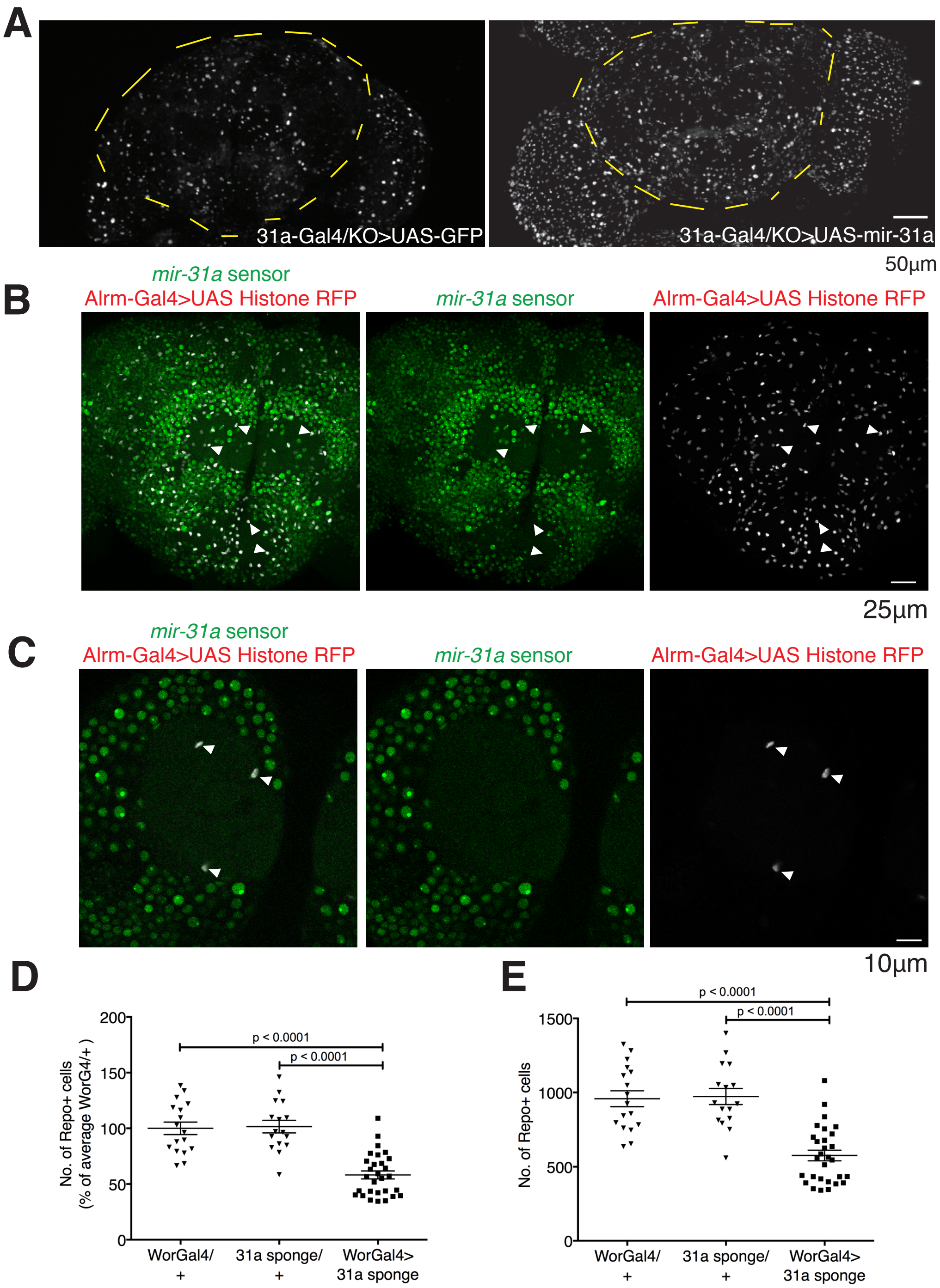

Appendix Figure S6

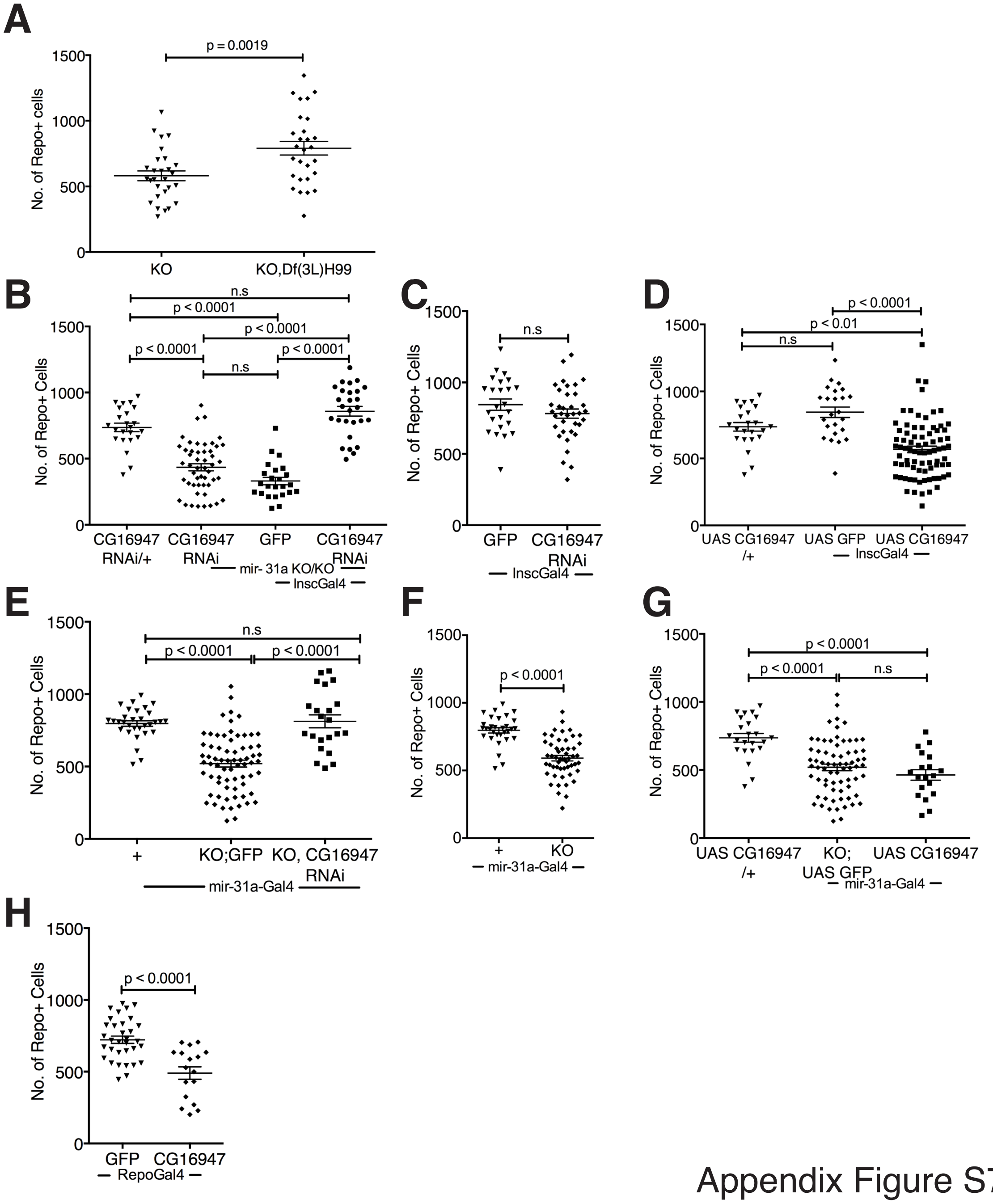

Appendix Figure S7

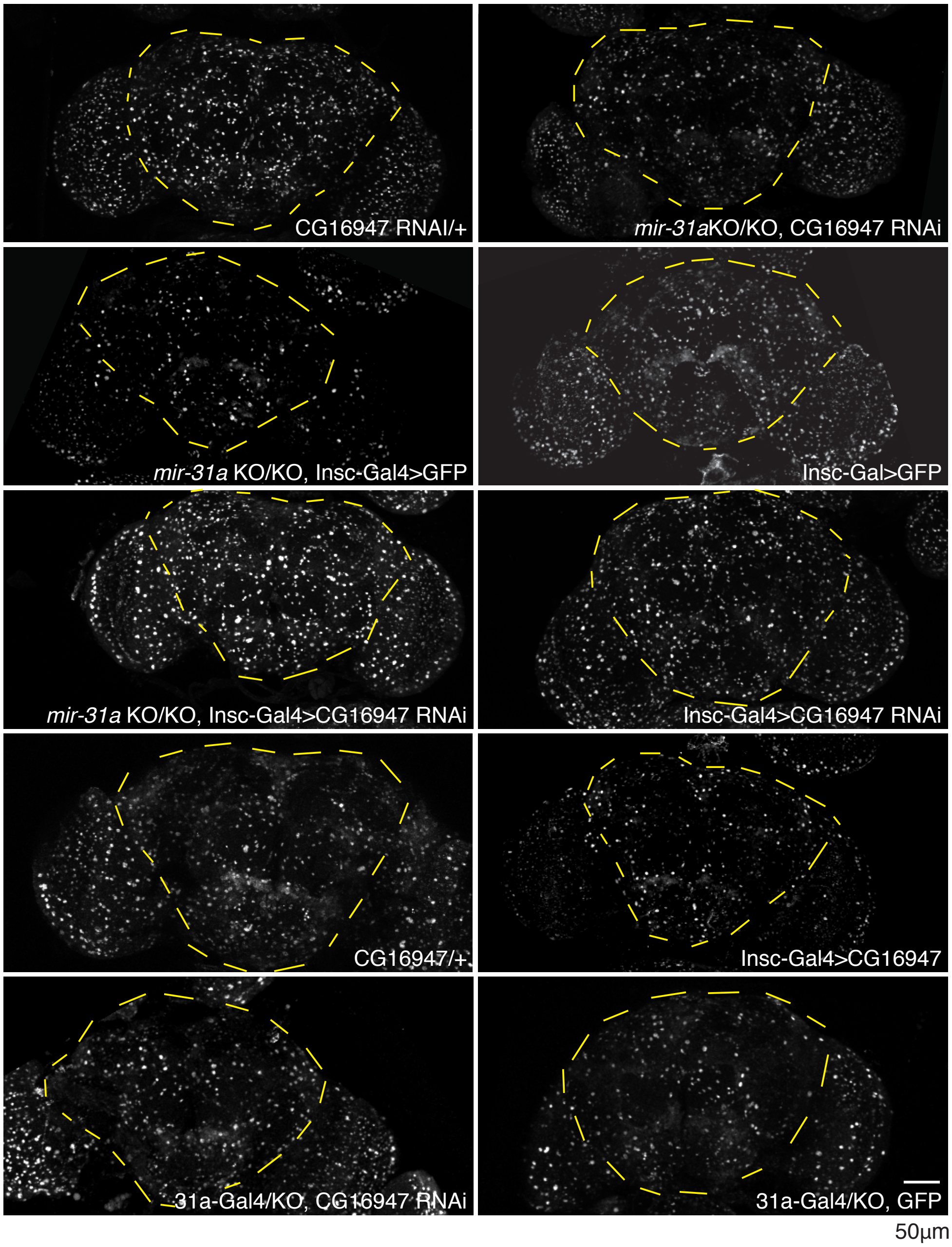

Appendix Figure S8

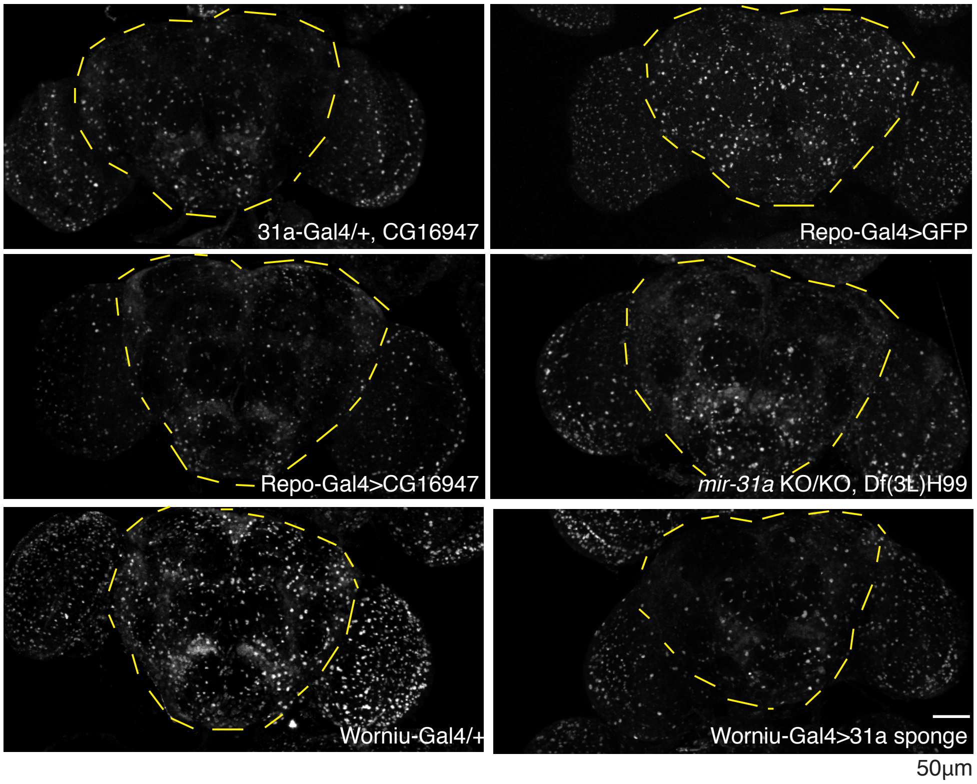

Appendix Figure S9

A

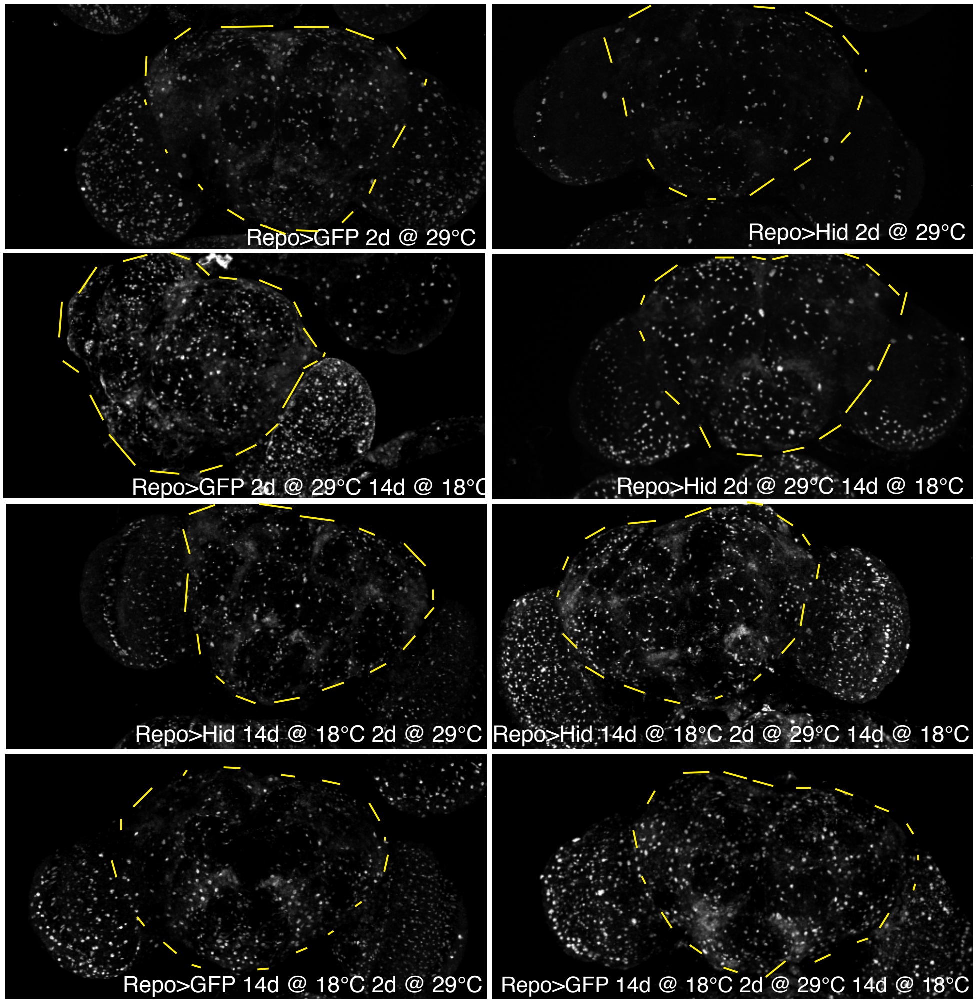

B

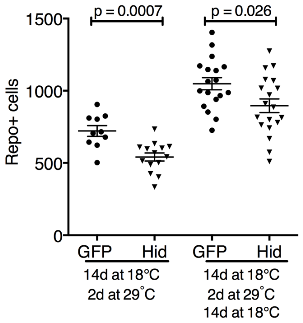

Appendix Figure S10
