# Supplementary material for: miR‐31 mutants reveal continuous glial homeostasis in the adult Drosophila brain
Source: EMBO J. 2017 Mar 20;36(9):1215–26. doi: 10.15252/embj.201695861 (PMC5412881; doi:10.15252/embj.201695861)
Supplement: Supplementary file 2 — Expanded View Figures PDF [file EMBJ-36-1215-s002.pdf]

# Expanded View Figures

**Figure EV1. *miR-31a* is expressed in adult progenitor cells that give rise to glia (related to Fig 1).**

A, B Number of glia at 2, 7 and 21 days post-eclosion represented as a percentage of the number in 2-day-old flies. Error bars represent SEM. Data were analysed using one-way ANOVA. (A) Canton S controls. (B) *miR-31a* mutants.

C Small significant difference in number of neurons in the central brain in 7-day-old adults was observed. Data are represented as a percentage of the average number of neurons in Canton S control animals. Data were quantified with Imaris (Bitplane). Unpaired Student's *t*-test was used for analysis. Error bars represent SEM.

D Astrocyte numbers (*Alrm-Gal4 > UAS-Histone-RFP*) in 7-days and 21-days controls (Ctrl) and *miR-31a* mutants (KO) represented as a percentage of the number in the CS controls. Unpaired Student's *t*-test was used for analysis. Error bars represent SEM.

E *miR-31a* sensor in a 2-days post-eclosion adult brain. *miR-31a* activity is indicated by the absence of GFP expression. White arrowheads point to example cells where GFP co-localizes with anti-repo (red), indicating low miRNA activity in the mature glia.

F *miR-31a* sensor (GFP) expression is excluded from some *Insc-Gal4 > UAS-Histone-RFP*-expressing cells (white arrowheads) in the brains of 2-days post-eclosion adults.

▶

© 2017 The Authors 

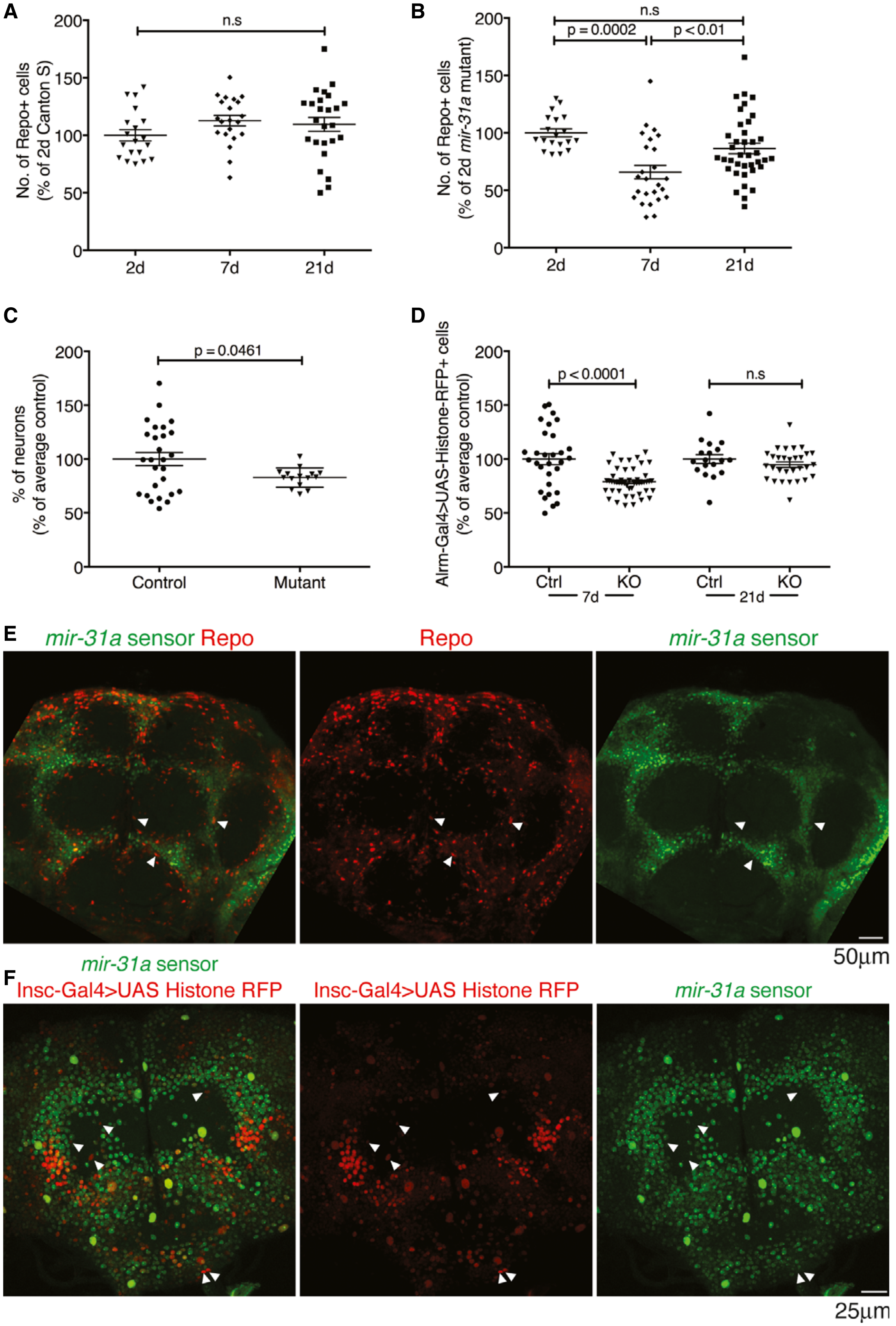


**Figure EV1.**

 © 2017 The Authors

**Figure EV2. Rchy1, the target of *miR-31a*, causes death of glia by apoptosis (related to Figs 2 and 3).**

A Left panel: number of repo-expressing and lineage-tagged $GFP^+$ cells. Right panel: lineage-tagged $GFP^+$ cells with active *repo-Gal4*. Data are presented as a ratio of the total number of GFP cells in the central brain. Error bars represent SEM.

B Number of glia in *miR-31a* mutants at 7 days with and without *Df(3L)H99*. Introducing *Df(3L)H99* prevented glial loss. Data were analysed with an unpaired Student's *t*-test. Error bars represent SEM.

C Alignment of fly and human Rchy1 proteins showing regions of sequence similarity. The antibody to human Rchy1 was raised against a peptide containing residues 87–167.

D The number of Rchy1-expressing cells in the central brain was greater in the *miR-31a* mutants than in Canton S controls (88 ± 0.4 vs. 17 ± 3). Data were analysed with an unpaired Student's *t*-test. Error bars represent SEM.

E Activated caspase-3-positive (green) glial cells stained with anti-repo (purple) are observed in the brains of 2-days post-eclosion adult *repo-Gal4* > *UAS-CG16947* brains. White arrowheads point to anti-repo-positive cells that are activated caspase-3-positive.

© 2017 The Authors

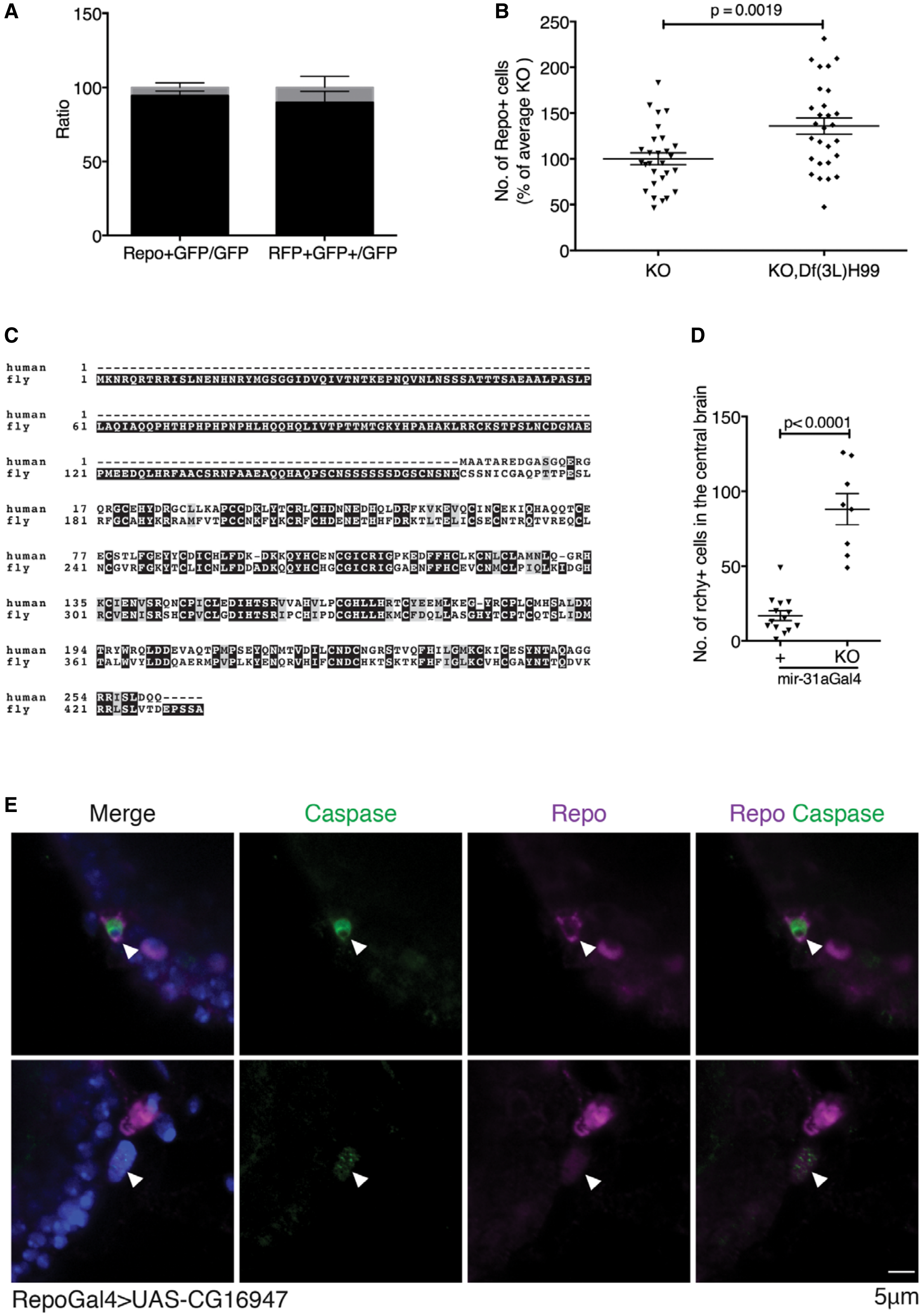


**Figure EV2.**

 © 2017 The Authors

**Figure EV3. Adult progenitor cells give rise to both neurons and glia (related to Figs 4 and 5).**

A *miR-31a-Gal4* stocks lacking FRT42D were crossed to *UAS-CD8-GFP,hsFlp;FRT42D, tubGal80* flies. Heat shock was induced 1 day post-eclosion and the flies dissected 1 day later. The bright green spots seen in the images are not correlated with DAPI, indicating that these are non-specific bright GFP spots. The control shows that the clones that we observe in the MARCM stocks are not phantom clones as no clones are observed in the no-FRT42D control.

B, C G-Trace [UAS-RFP, UAS-Flp, Ubi-p63E(FRT.Stop)GFP] lineage analysis of *miR-31a-Gal4*-expressing cells in the adult. Gal4 activity was limited to adult stages with Gal80$^{ts}$. RFP represents ongoing Gal4 activity (or recent activity allowing for perdurance). GFP is a permanent lineage tag for cells that have expressed Gal4. (B) Anti-elav was used to label neurons. Yellow arrowheads point to RFP$^{+}$ elav$^{+}$ cells. White arrowheads point to GFP$^{+}$ elav$^{+}$ cells. (C) Anti-repo was used to label glia. White arrowheads point to GFP$^{+}$ repo$^{+}$ cells.

D *Insc-Gal4*-expressing cells visualized with *UAS-Histone-RFP* gave rise to elav-expressing neurons (purple; white arrowheads) and repo-expressing glia (green; yellow arrowheads). Elav$^{+}$ RFP$^{+}$ and repo$^{+}$ RFP$^{+}$ cells represent non-overlapping populations.

E Images showing the central brain regions of flies carrying G-Trace [UAS-RFP, UAS-Flp, Ubi-p63E(FRT.Stop)GFP] with *Insc-Gal4* and *Gal80$^{ts}$*. Flies were reared at 18°C until eclosion. Flies that were not shifted to the permissive temperature of 29°C (left) showed very few GFP-expressing clones in the central brain. Flies that were reared right after eclosion for 7 days at 29°C had more clones than those that had been reared for only 1 day at 29°C.

F–H Flies carrying G-Trace with the *Insc-Gal4* driver and *Gal80$^{ts}$* were raised at 18°C until the indicated age, after which Gal4 was activated by shifting to 29°C for a further 7 days. Brains were labelled with anti-repo or anti-elav. RFP expression represents ongoing or recent Gal4 activity. GFP is a permanent lineage tag for cells that have expressed Gal4. Ctrl indicates the Canton S control background. KO indicates the *miR-31a* mutant background. (F) Images of brains shifted to activate Gal4 at 7, 14 and 21 days. Clones expressing repo and GFP are labelled with white arrowheads. (G) Cells expressing elav and RFP represented as a percentage of RFP-expressing cells. Although it appears that there may be a trend towards fewer elav-expressing cells in the earlier time points in the mutant brains, the scatter in the data is large, and the differences were not statistically significant. (H) Cells expressing repo and RFP represented as a percentage of RFP-expressing cells. The number of cells expressing repo was significantly increased in the mutant brains. Unpaired Student's *t*-test was used. Error bars represent SEM.

© 2017 The Authors

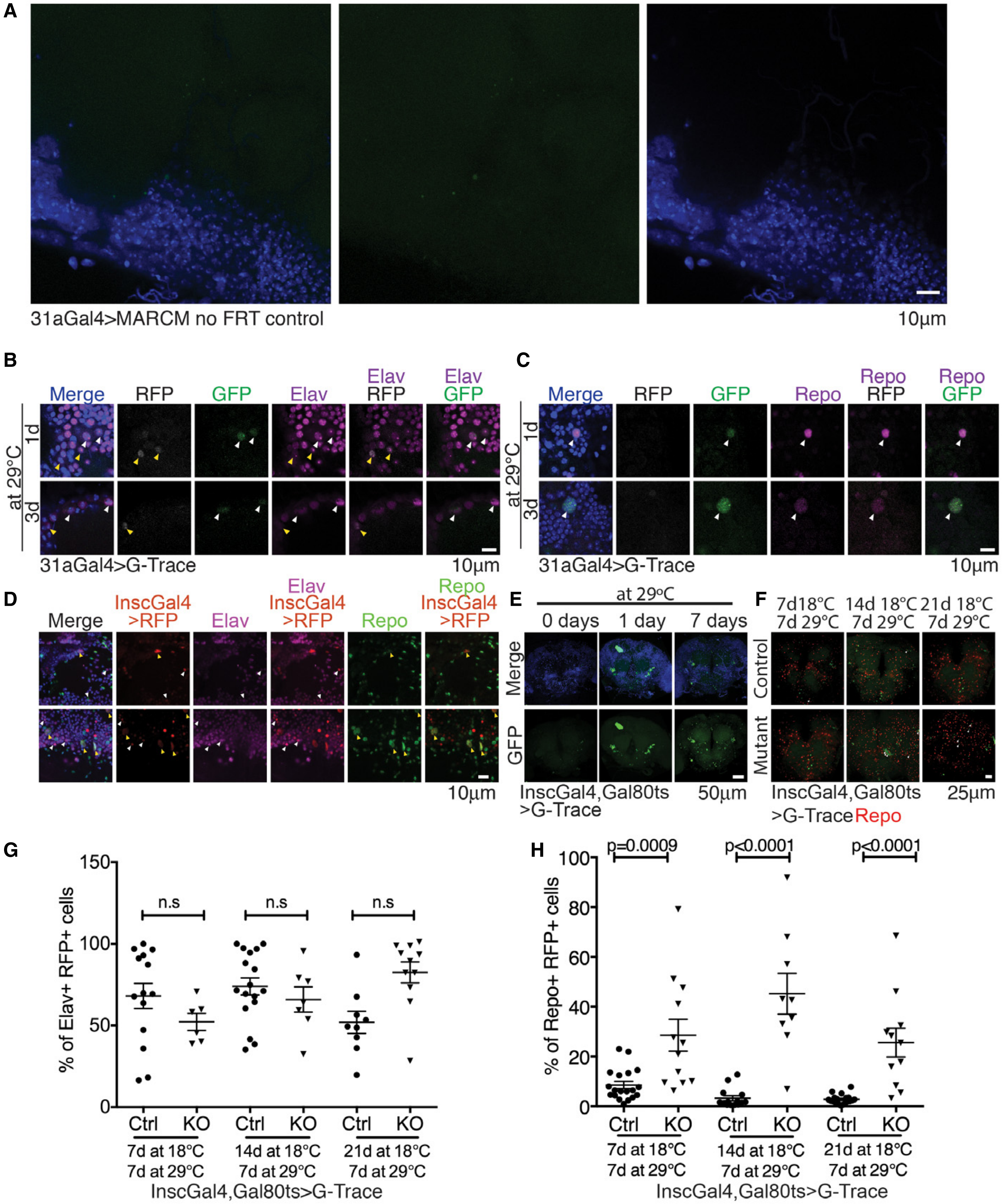


**Figure EV3.**


© 2017 The Authors

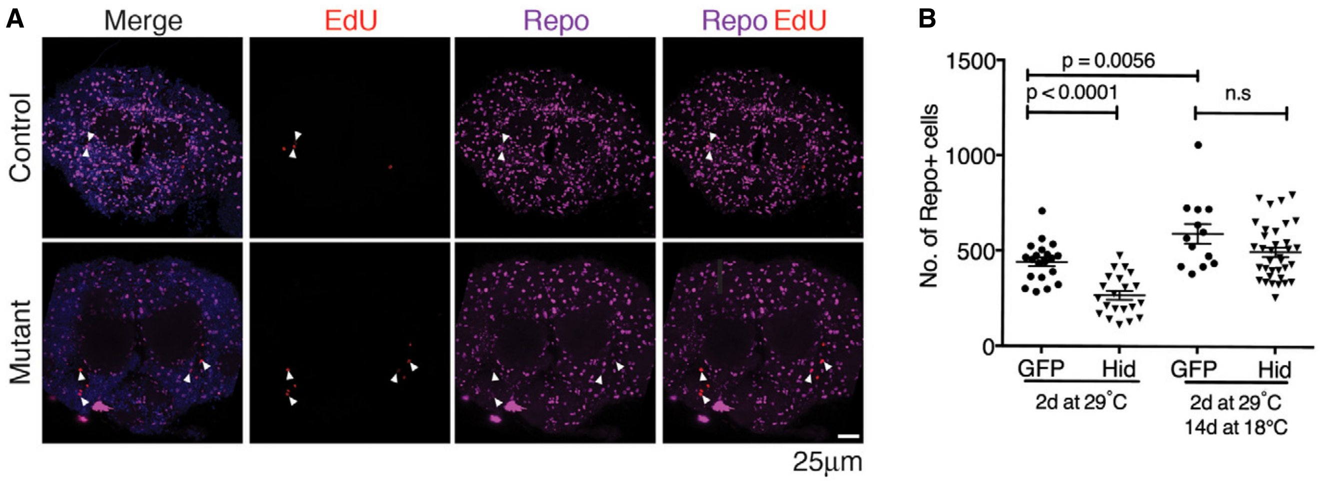


**Figure EV4. Active division of progenitor cells is observed in the adult brain of *Drosophila melanogaster* (related to Fig 5).**

A EdU-labelling experiment. Overview confocal stack images of EdU and repo immunoreactivity in the central brains of control and *miR-31a* mutant brains (mutant).

B Raw counts of anti-repo-positive glia in the central brain in glial ablation experiments where flies carrying *repo-Gal4* and $Gal80^{ts}$ were reared at 18°C until eclosion to keep Gal4 inactive. This was followed by 2 days at 29°C to express *UAS-hid* or *UAS-GFP* as a control. Left panels: flies were examined immediately after 2 days of transgene expression. Right panels: flies were allowed to recover for 14 days at 18°C before processing. Data were analysed using an unpaired *t*-test (two-tailed). ns: not significant. Error bars represent SEM.

© 2017 The Authors
